# Supplementary material for: Serum Sodium Concentration During Arginine Vasopressin Infusion in Critically Ill Children
Source: Children (Basel). 2024 Nov 9;11(11):1359. doi: 10.3390/children11111359 (PMC11592650; doi:10.3390/children11111359)
Supplement: Supplementary file 1 [file children-11-01359-s001.zip › children-3287012-supplementary.pdf]

Supplemental Material Table S1. Sensitivity Analysis for primary outcome (course of serum sodium before, during and after AVP treatment) of the 59 patients with complete data before, during, and after AVP treatment.

Variable	24h before AVP	during AVP	20-28h after AVP	p-value
n	59	59	59	
Sodium at AVP start (mmol/L)	NA	141.00 [137.0, 144.5]	NA	
Hyponatraemia at AVP start, n (%)	NA	9 (15.3)	NA	
130 to <135 mmol/L (mild)	NA	8 (13.6)	NA	
125 to <130 mmol/L (moderate)	NA	1 (1.7)	NA	
<125 mmol/L (severe)	NA	0 (0.0)	NA	
Mean serum sodium (mmol/L)	137.0 [134.0, 140.0]	138.2 [134.1, 142.5]	138.0 [136.0, 142.0]	0.343
Lowest serum sodium (mmol/L)	137.0 [134.0, 140.0]	135.0 [131.00, 140.00]	138.0 [136.0, 142.0]	0.010
Time to serum lowest sodium (h)	NA	14.7 [1.6, 32.4]	NA	
Hyponatraemia, n (%)	16 (27.1)	27 (45.8)	12 (20.3)	0.008
130 to <135 mmol/L (mild)	14 (23.7)	16 (27.1)	10 (16.9)	
125 to <130 mmol/L (moderate)	2 (3.4)	8 (13.6)	2 (3.4)	
<125 mmol/L (severe)	0 (0.0)	3 (5.1)	0 (0.0)	

Continuous variables are presented as median [interquartile range], categorial variables are presented as number (percent).

At AVP treatment start the serum sodium concentration for 59 study patients was 141 mmol/L [137.00-144.50] with hyponatraemia already present in 9/59 (15.3 %) of patients. Mean serum sodium concentration during AVP therapy was 138.2 mmol/l [134.1-142.5]. During AVP treatment, the lowest sodium concentration (nadir: 135 mmol/L [131-140]) was observed 14.7 h [1.6-32.4] after start of AVP treatment, 27/59 (45.8 %) of patients experienced hyponatraemia at lowest sodium concentration during AVP treatment (among them moderate hyponatraemia in 8/59 (13.6 %) of patients, severe hyponatraemia (<125 mmol/L) in 3 (5.1 %) of patients). The incidence of hyponatraemia differed significantly before, during, and after AVP treatment (P=0.008), serum sodium concentrations <135 mmol/L were observed in 16/59 (27.1 %) of patients 24 h prior to AVP start and in 12/59 (20.3 %) 20-28 h after AVP treatment stop. Correspondingly, also lowest sodium values differed significantly before, during, and after AVP treatment (P=0.010), sodium concentrations 24h prior to AVP start were 137 [134.0-140.0] mmol/L, and 20-28 h after AVP treatment stop were 138 mmol/L [136.0-142.0].
